# Incidence of aneuploidy in embryos studied using PGT-A in Mexico: a clinical retrospective experience

**DOI:** 10.61622/rbgo/2025rbgo87

**Published:** 2025-11-18

**Authors:** Eduardo Ponce-Najera, Diana María Ojeda-López, Rodolfo González-Holveman, Ricardo Rodríguez-Calderon, Karla Patricia Bedia-Mejía, Jose Carlos Salazar-Trujillo, Jorge Luis Lezama-Ruvalcaba, Carlos Gerardo Salazar López-Ortiz

**Affiliations:** 1 Hospital Español de México Assisted Reproduction Clinic HISPAREP Mexico City Mexico Hospital Español de México, Assisted Reproduction Clinic HISPAREP, Mexico City.

**Keywords:** PGT-A, Aneuploidy, Maternal age, Genetic testing, Fertilization in vitro, Preimplantation diagnosis, Reproduction, High-throughput nucleotide sequencing

## Abstract

**Objective:**

This study aimed to analyze the relationship between aneuploidy and maternal age based on preimplantation genetic testing for aneuploidies (PGT-A) and the distribution of aneuploidy across individual chromosomes.

**Methods:**

This is a single-center retrospective cohort study. The study included patients who underwent PGT-A during their in vitro fertilization (IVF) cycle between 2016 and 2024. PGT-A was performed on 1,341 embryos from 481 patients using next-generation sequencing (NGS)–based techniques. We conducted a Logistic regression analysis to determine the relationship between maternal age and the rate of aneuploidy; additionally, the most commonly affected chromosome and the characteristics of the detected alterations were evaluated. Statistical analysis was performed using STATA v17.

**Results:**

Among all patients, the observed aneuploidy rate was 55.85%. In patients younger than 30 years, the aneuploidy rate was 26.92%, with a progressive increase to 62% in women older than 40 years. Chromosomes 22, 21, 16, and 15 were the most frequently affected. Logistic regression indicated that age is a significant predictive factor for aneuploidy (coefficient = 0.0956; p < 0.001), with an odds ratio of 1.10 (95% CI: 1.07–1.13), meaning that the probability of aneuploidy increases by approximately 10% for each additional year of age.

**Conclusion:**

Our results indicate that an increase in maternal age is significantly associated with a higher incidence of aneuploidy in embryos that underwent PGT-A. These findings are significant for patient counseling and optimizing embryo selection in assisted reproduction treatments.

## Introduction

The frequency with which older women seek pregnancy in Europe and North America has steadily increased over the past decades.^(1)^ In Latin America, the trends are beginning to look similar.^(2)^ The oocyte quality declines as a woman ages, leading to decreased fecundity.^(3)^ This is primarily due to chromosomal anomalies that can arise at any stage of cell division and are considered a common phenomenon.^(4)^ Between 40% and 50% of blastocyst-stage embryos are estimated to present some chromosomal abnormality.^(5)^ Among these abnormalities, aneuploidy is one of the main barriers to achieving successful pregnancies in in vitro fertilization (IVF) treatments.^(6)^ This type of alteration is generally the result of errors during maternal meiotic divisions.^(7)^

In the beginning, embryo selection was based solely on microscopic morphological criteria, which do not directly correlate with the embryo's actual genetic status.^(8)^ Nowadays, advanced methods such as comparative genomic hybridization microarrays (aCGH) and next-generation sequencing (NGS) allow for a precise evaluation of the entire embryonic genome through trophectoderm biopsy.^(9)^

Preimplantation genetic testing for aneuploidies (PGT-A) is a technique that permits the analysis of the embryo's genetic component. Its primary objective is to facilitate the selection and transfer of euploid embryos, which has been associated with a significant increase in implantation rates, clinical pregnancies, and live births, especially in patients older than 35.^(10)^

This study retrospectively analyzed the PGT-A results of ART cycles in our center to assess the incidence of aneuploidy, characterize the types and chromosomal distribution of the aneuploidies observed, and examine the relationship between maternal age and aneuploidy rates.

## Methods

We conducted a retrospective analysis of 1,341 embryos from couples who underwent in vitro fertilization with preimplantation genetic testing for aneuploidies (PGT-A) at our reproduction center in Mexico between January 2016 and December 2024. A total of 481 patients were included, each of whom presented with at least one of the following conditions: advanced reproductive age (≥35 years), recurrent implantation failure (defined as two or more unsuccessful IVF cycles), recurrent miscarriage (two or more pregnancy losses), or male factor infertility. All patients elected to proceed with PGT-A. Cases involving donor gametes or those with previously identified chromosomal abnormalities were excluded. The data obtained from the electronic files is entirely anonymous; a database was filled with exclusive access for the researchers. The ethics committee approved this study.

We based our descriptive analysis of the PGT-A results on the work performed by Nair et al.^(11)^ to characterize in detail the chromosomal abnormalities present in the studied embryos. This analysis included evaluating the frequency of errors in individual chromosomes, the distribution of monosomies and trisomies for each chromosome, the incidence of aneuploidies in relation to maternal age, the total number of affected chromosomes, and the total number of aneuploid embryos in each age group.

A univariate logistic regression analysis was also performed to assess the relationship between maternal age and the likelihood of an embryo presenting aneuploidy. This analysis allowed estimation of the odds ratio (OR) per additional year of age, providing a quantitative measure of the increase in the probability of chromosomal aberrations associated with maternal aging, with a significance level set at 0.05. Statistical analysis was carried out using STATA v17.

## Results

**Figure 1:**
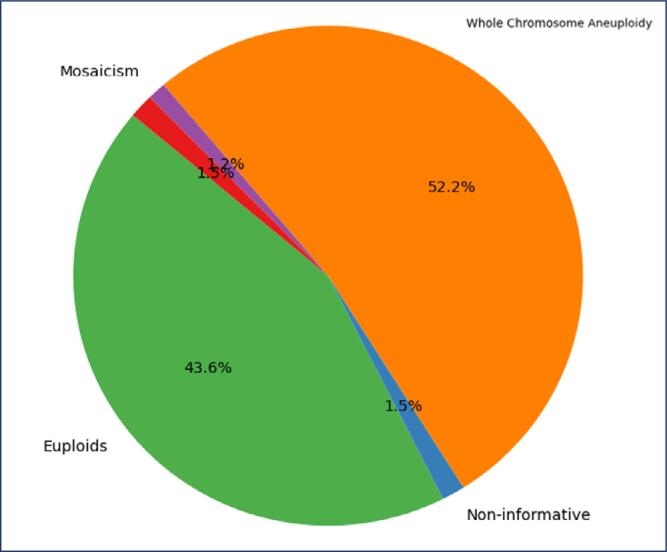
Distribution of evaluated embryos

A total of 1,341 blastocyst-stage embryos from 481 patients of 20 to 48 years of age (mean age of 37.73 ± 4.31 years). Of the 1,341 embryos evaluated, 592 (44.15%) were classified as euploid, while 710 (52.95%) presented whole chromosome aberrations (Whole Chromosome Aneuploidy). Additionally, 16 embryos (1.19%) showed mosaicism, 21 embryos (1.57%) were considered non-informative, and two embryos (0.15%) exhibited segmental aberrations combined with whole chromosome aberrations (Segmental + Whole Chromosome Aneuploidy) (Figure 1).

### Distribution of aneuploidy detected by chromosome

The frequency of aneuploidy among the chromosomes is shown in figure 2a. The frequency of a particular chromosome exhibiting monosomy and trisomy is displayed in figure 2b. Aneuploidy was most frequently observed in chromosomes 22, 21, 16, and 15. Trisomies were more common than monosomies: trisomy 22 was the most common (n=56; 7.1% of all trisomies), followed by trisomy 21 (n=54; 6.9% of all trisomies) and trisomy 16 (n=44; 5.6%).

**Figure 2a:**
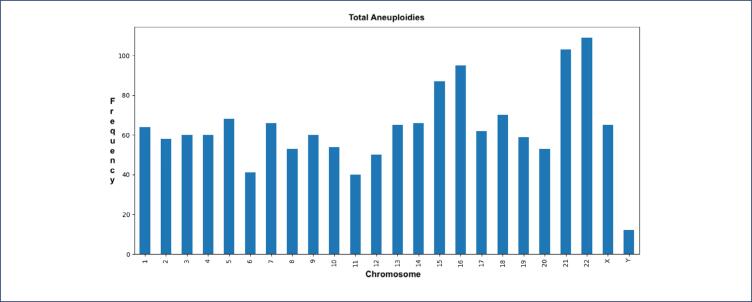
Frequency of aneuploidy among chromosomes

**Figure 2b:**
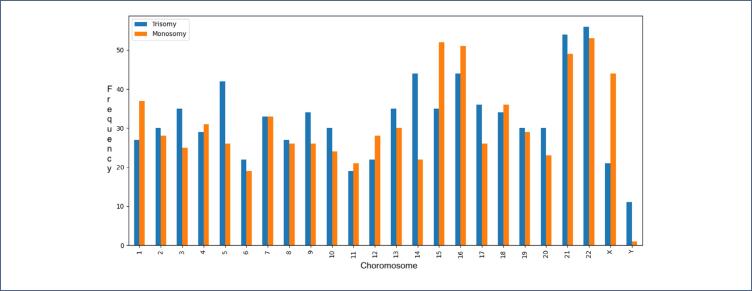
Frequency of a particular chromosome exhibiting monosomy and trisomy

### Aneuploidy with advanced maternal age

The types of chromosomal errors were analyzed and divided by age group. Maternal age at the time of trophectoderm biopsy was grouped as follows: <30, 31–34, 35–37, 38–40, and >40 years. The prevalence of whole chromosome aneuploidy among age groups is depicted in Figure 3a. The rate of whole chromosomal aneuploidy was 26.98% in women under 30 years old and increased to 62% in women over 40 years old (Figure 3a).

**Figure 3a:**
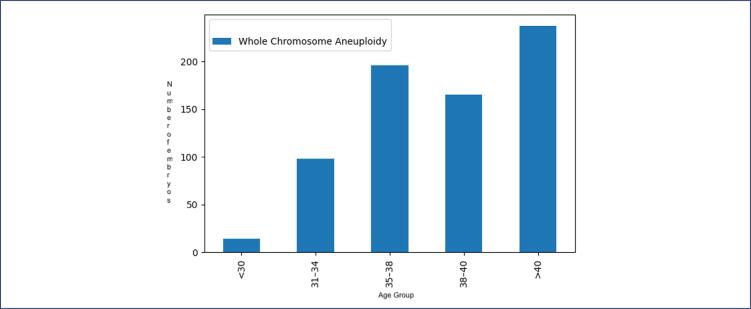
Prevalence of whole chromosome aneuploidy among age groups

Figure 3b shows the complexity of the chromosomal alterations. The number of chromosomal errors found in women over 40 affected a higher percentage of embryos with two, three, or more affected chromosomes compared to aneuploid embryos from women under 30 (Figure 3b).

**Figure 3b:**
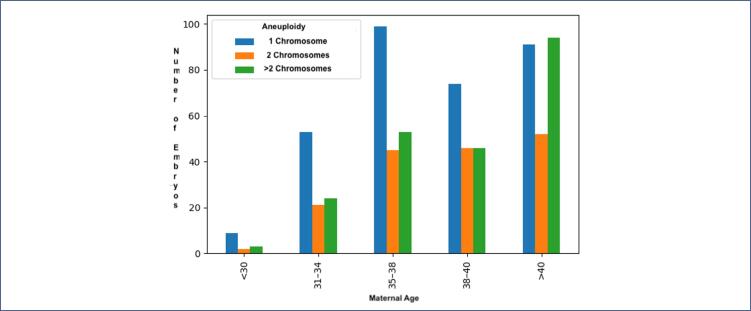
Complexity of the chromosomal alterations

**Figure 4:**
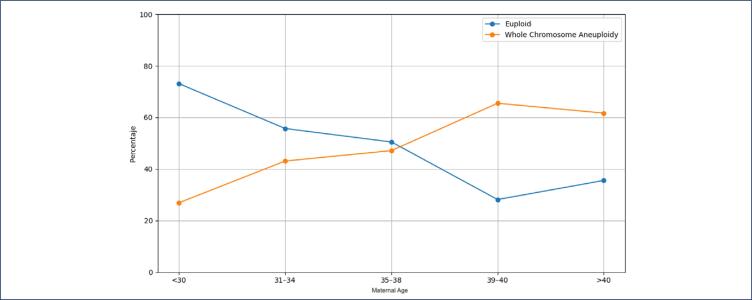
Relationship between maternal age and embryo chromosomal status, showing a progressive decline in euploid embryos and a corresponding increase in whole chromosome aneuploidy

**Figure 5:**
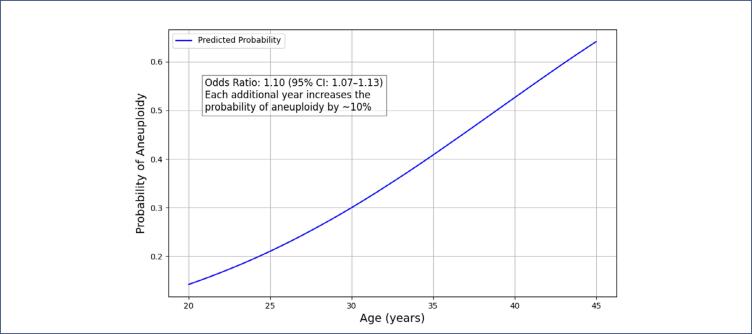
Effect of maternal age on the probability of embryo aneuploidy

The effect of age on the probability of an embryo being aneuploid was evaluated using univariate logistic regression analysis (Figures 4 and 5). The results indicated an odds ratio of 1.10 (95% CI: 1.07–1.13). This means that for each additional year, the probability of an embryo being aneuploid increases by approximately 10%.

## Discussion

The incidence of embryonic aneuploidy is one of the significant challenges in assisted reproductive treatments, with its prevalence increasing markedly with advanced maternal age.^(3)^ In general IVF populations, the percentage of chromosomally abnormal embryos rises from approximately 30–40% in women under 35 years to over 50% in women above 40 years.^(12)^ This strong maternal age effect reflects, in large part, the increased frequency of meiotic errors in aging oocytes, likely due to prolonged arrest in prophase I and gradual deterioration of the meiotic apparatus.^(7)^ For instance, Franasiak et al.^(12)^ observed an increasing percentage of embryos with abnormalities in more than one chromosome with advanced maternal age, highlighting a greater complexity of chromosomal errors in patients aged 40 years and above. Clinically, these findings translate into lower implantation rates and a higher risk of pregnancy loss in older patients due to the reduced availability of euploid embryos.^(13)^

Several studies employing preimplantation genetic testing for aneuploidies (PGT-A) have demonstrated that smaller autosomes are more prone to numerical aberrations.^(14)^ In particular, chromosomes 16, 22, 21, and 15 are most frequently affected.^(11,14)^ For instance, Liu et al.^(15)^ analyzed over 1,000 embryos using next-generation sequencing (NGS). They found that these four chromosomes accounted for a large proportion of the detected aneuploidies (trisomies or monosomies), consistent with our study.^(15)^ There is notable concordance in these trends when comparing findings from Latin American populations with those from Europe and the United States.^(1,2,16)^

Our results in Mexico align with international reports regarding the influence of maternal age and the chromosomal patterns of aneuploidy.^(10,12,13)^ Although there has historically been a scarcity of published data from Latin America, recent evidence suggests that the region exhibits aneuploidy rates similar to those of other areas when controlling for maternal age; for instance, a Colombian study found that age-stratified aneuploidy rates were equivalent to those reported in European cohorts.^(17)^ Similarly, Kotdawala et al.^(16)^ compared Spanish and Indian patients undergoing PGT-A and found no significant differences in the proportion of aneuploid embryos when matched by maternal age and clinical indications. Moreover, an analysis in the United States that evaluated the impact of genetic ancestry using ancestry-informative markers confirmed that the rate of embryonic aneuploidy does not significantly differ among women of European, Asian, African, or Latino descent when adjusted for maternal age.^(18)^ These observations support the idea that the effect of maternal age on embryonic euploidy is a universal biological phenomenon, regardless of population differences.

## Conclusion

Recent scientific literature reinforces that advanced maternal age is strongly associated with an increased incidence of embryonic aneuploidies across all studied populations and that the chromosomal patterns—particularly alterations in chromosomes 15, 16, 21, and 22—are highly consistent among Mexican/Latin American patients and data from Europe and North America. Our findings align with the international landscape, emphasizing their clinical relevance in our region. The evidence suggests that implementing PGT-A in older patients in our setting, as elsewhere in the world, is important for detecting and avoiding the transfer of aneuploid embryos, potentially improving reproductive outcomes in this high-risk group. However, given the retrospective design and the clinical indications guiding PGT-A in our center (advanced maternal age, recurrent implantation failure, recurrent miscarriage, or male factor infertility), there is potential for selection bias that could affect the observed aneuploidy rates. Because patients undergoing PGT-A represent a specific subset of the IVF population, the findings may not be fully generalizable to unselected IVF cohorts. Future research with prospective designs and broader inclusion criteria in diverse patient populations would help to confirm and refine these observations, mitigating indication-related biases.
